# Multi-Omics Analysis Reveals Disturbance of Nanosecond Pulsed Electric Field in the Serum Metabolic Spectrum and Gut Microbiota

**DOI:** 10.3389/fmicb.2021.649091

**Published:** 2021-07-02

**Authors:** Yeping Dong, Jiahua Lu, Ting Wang, Zhiliang Huang, Xinhua Chen, Zhigang Ren, Liangjie Hong, Haiyu Wang, Dezhi Yang, Haiyang Xie, Wu Zhang

**Affiliations:** ^1^Shulan (Hangzhou) Hospital Affiliated to Zhejiang Shuren University, Shulan International Medical College, Hangzhou, China; ^2^Shulan International Medical College, Zhejiang Shuren University, Hangzhou, China; ^3^Division of Hepatobiliary and Pancreatic Surgery, Department of Surgery, The First Affiliated Hospital, School of Medicine, Zhejiang University, Hangzhou, China; ^4^Key Laboratory of Combined Multi-Organ Transplantation, Ministry of Public Health, Hangzhou, China; ^5^Institution of Organ Transplantation, Zhejiang University, Hangzhou, China; ^6^Department of Infectious Diseases, The First Affiliated Hospital of Zhengzhou University, Zhengzhou, China; ^7^Department of Polymer Science and Engineering, Institute of Biomedical Macromolecules, Zhejiang University, Zhengzhou, China

**Keywords:** nanosecond pulsed electric field, liver ablation, lipid metabolism, metabonomics, gut microbiota

## Abstract

Nanosecond pulsed electric field (nsPEF) is a novel ablation technique that is based on high-intensity electric voltage to achieve tumour-killing effect in the target region, and increasingly considered for treating tumours of the liver, kidneys and other organs with rich blood supply. This study aims to observe effect of nsPFE treatment on serum metabolites and gut microbiota. The serum and faecal specimens of the pigs were collected pre- and post-treatment. The gut microbiota of pigs was sequenced by Illumina Miseq platform for analysing the diversity and alterations of gut microbiota. Liquid chromatography-mass spectrometry (LC-MS)-based metabonomic analysis and Pearson coefficient method were also used to construct the interaction system of different metabolites, metabolic pathways and flora. A total of 1,477 differential metabolites from the serum were identified by four cross-comparisons of different post-operative groups with the control group. In addition, an average of 636 OTUs per sample was detected. Correlation analysis also revealed the strong correlation between intestinal bacteria and differential metabolites. The nsPEF ablation of the liver results in a degree of liver damage that affects various metabolic pathways, mainly lipid metabolism, as well as gut microbiota. In conclusion, our study provided a good point for the safety and feasibility of applying nsPEF on liver through the integrated analysis of metabolomics and microbiomes, which is beneficial for the improvement of nsPEF in clinical use.

## Introduction

Nanosecond pulsed electric field (nsPEF) is a novel electric power-based ablative technique for treating a variety of tumours in solid organs including liver, kidneys, pancreas, prostate, etc. When applied with appropriate parameters, nsPEF will not lead to heat-based cytotoxicity, meaning non-thermal (Pliquett and Nuccitelli, 2014). Thus, nsPEF can achieve tumour-killing effect and simultaneously avoid thermal injury to the adjacent vessels or organs in target regions. Compared with conventional heat-based ablation techniques such as radiofrequency ablation (RFA) or microwave ablation (MWA), nsPEF does not directly cause Joule heating in the target ablation area. Instead, it induces apoptosis of tumour cells and inhibits tumour angiogenesis. As of today, nsPEF has been widely used to treat a great number of cancers, including malignant melanoma (Chen et al., 2010; Rossi et al., 2019; Zhang et al., 2019), skin basal cell carcinoma (Yang et al., 2011), lung squamous cell cancer (Hornef et al., 2020), as well as skin squamous cell carcinoma (Yin et al., 2012). Previous studies have reported the treatment of HCC with nsPEF in animal models since nsPEF does not cause direct Joel heating but induces complete cancer cell death (Yin et al., 2014; Xu et al., 2018). When we were looking at the safety and feasibility of applying nsPEF ablation in porcine livers, we found that the indicators of liver function after ablation fluctuated. The elevated levels of lactate dehydrogenase (LDH), aspartate aminotransferase (AST), and alanine aminotransferase (ALT), suggest possible hepatocyte injury. Since the liver is an important metabolic organ, it is worth discussing whether there are metabolic changes in pigs after nsPEF treatment.

Metabolomics is an integral part of systems biology, which makes qualitative and quantitative analyses of all kinds of metabolites and endogenous metabolites of organisms when they are stimulated or disturbed and finds out the relative correlation between metabolites and physiological and pathological changes in organisms (Deda et al., 2017; Law et al., 2017). Metabolites, especially a combination of multiple small-molecule metabolites could be a promising tool for the diagnosis and prognosis of diseases (Cheng et al., 2015). Its research object is mostly small molecular metabolites including substrates and products of various metabolic pathways (MW < 1,000). Serum is often the source for metabolic profiling because it is considered a pool of metabolites (Sreekumar et al., 2009). Liquid chromatography-mass spectrometry (LC-MS) provides good reliability and reproducibility in the detection of small molecular metabolites. To further study the influence of the nsPEF in the treatment of liver, we collected serum of pigs pre-and post-treatment for LC-MS-based metabolomic analysis.

However, a single study on metabolomic analysis cannot fully and systematically elucidate the influence of the nsPEF in the treatment of liver. Due to the emergence and development of metabonomics, the one-sided idea that host metabolism is only controlled by its own gene expression has been gradually corrected. Host metabolism is jointly regulated by its own genes and the symbiotic microbial genome *in vivo* (Aleksandrova et al., 2017). The metabolism of substances cannot be completed independently by the host, which requires the joint action of gut microbiota. Under physiological state, the liver can remove toxins from the intestines, such as endotoxins, ammonia, indoles, phenols, pseudo-neurotransmitter precursors, short-chain fatty acids and intestinal bacteria, fungi, etc. So the intestinal microecology can change significantly when the liver is damaged. Over the years, a great deal of studies have revealed the important roles of intestinal bacteria, or in another word, gut microbiota, in the development of cardiovascular diseases (Kitai and Tang, 2017; Tang et al., 2017), obesity (Israel, 2015), chronic liver diseases (Wieland et al., 2015), and tumour (Mendizabal and Silva, 2016). In addition, it was reported that the gut microbiota was altered significantly in the progression of liver disease, even early injury (Xie et al., 2016). Therefore, we also combined 16S rRNA technology to analyse the connection of metabolomics and gut microbiota. 16S rRNA technology is a high-throughput assay for microorganisms based on metagenomics and is often used to detect the diversity sequence of gut microbiota. Therefore, this study intends to adopt LC-MS-based metabolomic analysis of serum samples, combined with the 16S rRNA technology of the Illumina MiSeq platform to explore the safety and feasibility of applying nsPEF in porcine livers from the perspective of the correlation between metabolites, pathways, and gut microbiota.

## Materials and Methods

### Animal Care and Ethics

There are great similarities between pigs and humans in physiological anatomy, nutrient metabolism, biochemical indexes, and other characteristics. In order to get closer to clinical practice and facilitate anaesthesia monitoring, blood sampling, ultrasound-guided puncture, and other operations, a total of 10 male Yorkshire pigs (45–55 kg; mean, 50 kg; 3–4 months) were purchased and maintained by the Animal Research Centre of Zhejiang Chinese Medical University Laboratory. Appropriate human care of all pigs was taken by experienced laboratory breeder, and the pigs were provided with the same formula diet and clean water under laboratory conditions. Treatment protocols with nsPEF was approved by The Animal Care and Use Committee of Zhejiang Chinese Medical University (certificated approval number IACUC-20191216-04), and the procedures were carried out following the guidance from the State Council of the People’s Republic of China (Decree No. 2, 1988) for the care and use of experiment animals.

### Anaesthesia and Interventional Procedures

General anaesthesia was maintained with 1.5% to 2% isoflurane. All pigs were treated with a neuromuscular blockade to ensure complete muscle paralysis. A two-needle electrode with effective tip length of 2 cm was placed around the major hepatic veins with colour ultrasound guidance.

### Procedures of Nanosecond Pulsed Electric Field Ablation

The ablation treatment was carried out with a pulse generator device with two electrodes developed by Ready Biological Technology Corporation (Hangzhou, China). The applied parameters of nsPEF including pulse number: 500 pulses, electric voltage: 25 kV, the pulses were delivered at the absolute myocardial refractory period [each pulse after the R-wave on the electrocardiograph (ECG)] for the prevention of heart arrhythmias. The pulse frequency was set as 0.5 Hz. The spacing distance between the two electrodes was 1.5 cm for each ablation lesion. Three nsPEF lesions were produced per pig and targeted directly adjacent to the hepatic veins at the central area of porcine livers (Supplementary Figure 1).

### Collection and Preparation of Samples

Blood samples were collected from the auricular veins 1 h prior to nsPEF treatment for identification of baseline parameters. After nsPEF treatment, blood samples were collected periodically (at 1 h, 1 day, 3 days, and 7 days post-treatment) to monitor liver function. The blood sample was centrifuged once for 15 min at 3,000 rpm at room temperature, and the supernatant was divided into two parts. One part was measured by automatic biochemical analyser to reflect the liver function injury. The other part was stored in the refrigerator at −80°C for metabolomic analysis.

Faecal samples were collected 1 h pre-treatment, 1 h, 1 day, 3 days, and 7 days post-treatment. The contents at the end of the cecum were placed in a sterile centrifuge tube, frozen in liquid nitrogen, and were stored at an ultra-low temperature of −80°C for the analysis of gut microbiota diversity.

### Biochemical Assay

Serum levels of alanine aminotransferase (ALT), aspartate aminotransferase (AST), and lactate dehydrogenase (LDH) were measured by the respective commercially available testing kits (Nanjing Jiancheng Bioengineering Institute, China), according to the manufacturer’s instructions. After adding the colour solution 2,4-dinitrophenylhydrazine, the pre-treated sample was treated in a water bath at 37°C for 20 min and then the OD value was measured. The biochemical assay was processed in the Animal Research Centre of Zhejiang Chinese Medical University Laboratory.

### Serum Metabolic Profiling

Fifty serum samples (10 for each time point) stored at −80°C were thawed on ice and then dissolved in methanol for internal standardisation. After homogenisation and purification, the supernatants were resolved in water and methanol and transferred to LC vials pending analysis. ACQUITY UPLC I-Class system (Waters Corporation, Milford, MA, United States) combined with VION IMS QTOF Mass spectrometer (Waters Corporation, Milford, MA, United States) was used for the untargeted metabolic profiling in either ESI-positive or ESI-negative ion modes. An ACQUITY UPLC BEH C18 column (1.7 μm, 2.1 × 100 mm) was employed in either mode. All the samples were kept on ice during the procedure, and the injection volume was 1 μl. Water and acetonitrile/methanol 2/3 (*v*/*v*), both containing 0.1% formic acid were used as mobile phases A and B, respectively. (Linear gradient: 0 min, 5% B; 1 min, 5% B; 12 min, 100% B; 16 min, 100% B; 16.1 min, 5% B; 18 min, 5% B.) Data acquisition was performed in full-scan mode (*m*/*z* ranges from 70 to 1,000) combined with MSE mode. (Spray voltage, 3.8 kV; capillary temperature, 320°C; Aux gas heater temperature, 350°C; sheath gas flow rate, 35 Arb, and Aux gas flow rate, 50 Arb).

### Gut Microbiota Analysis

Faecal samples of the pigs were collected upon defecation and immersed in liquid nitrogen immediately. TIANamp Stool DNA Kit (Tiangen, Beijing, China) was used to extract gut microbial DNA from faecal samples according to the manufacturer’s protocols. The concentration and integrity of DNA was verified using a NanoDrop (Thermo Fisher Scientific, Wilmington, DE, United States).

Purified amplicons were performed on an Illumina MiSeq platform according to the standard protocols by Shanghai Itechgene Technology, China. Raw sequencing data were edited and compiled into FASTQ format. Trimmomatic software19 was then used to pre-process paired-end reads from the original DNA fragments to detect and trim ambiguous bases. Paired-end reads were assembled into tags according to unique barcodes using FLASH software20 after careful trimming. Clean sequences were removed by primer sequence and clustered for the generation of operational taxonomic units (OTUs) using Vsearch software with 97% identity. Total representative sequences for OTUs were annotated and blasted against the Silva database with the RDP classifier program (confidence threshold was 70%).

### Data Analysis

The proqenesis QI software (Waters Corporation, Milford, MA, United States) was used to collect and analyse the LC-MS raw information for feature alignment, signal integration, and normalisation to construct a 3D-data matrixes containing retention time-*m*/*z* pairs, peak intensities, and sample information. Metabolites were then identified and annotated by progenesis QI (Waters Corporation, Milford, MA, United States) Data Processing Software, based on MS/MS fragmentation data from the public databases including HMDB and LipidMaps database. The internal standard was adopted for data QC. We then evaluated the pre-processed data using a strategy combining multivariate and univariate methods. Supervised orthogonal partial least square-discriminant analysis (OPLS-DA) was used to discriminate the serum metabolites that were altered after the analysis between the two groups of samples. The validation of OPLS-DA models was performed following the sevenfold cross-validation process. Variable importance in the projection (VIP) value was used to rank the contribution of each variable for the established OPLS-DA model, and variables with VIP value > 1 are of significance for group discrimination. The differentially expressed metabolites were selected by combining the VIP value threshold (VIP > 1) obtained by the OPLS-DA model with *p*-values (*p* < 0.05) from a two-tailed Student’s *t*-test on the normalised peak areas.

## Results

**FIGURE 1:**
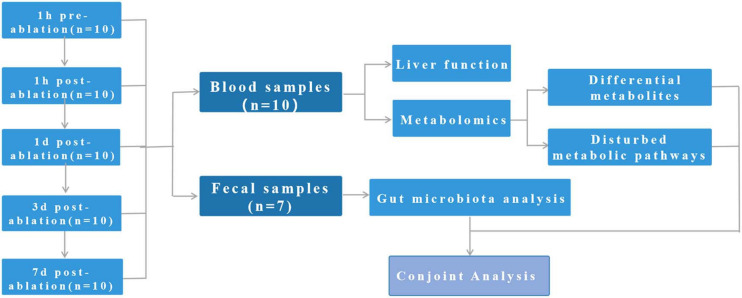
Study design: This study involves 10 pigs. Blood samples were collected from all 10 pigs at all time points, and faecal samples were completely collected from seven of the pigs.

This study involves 10 pigs. Blood samples were collected from all 10 pigs at all time points, and faecal samples were completely collected from seven of the pigs (Figure 1). All pigs survived the procedure, and their vital signs remained stable throughout the treatment period.

### Liver Function

**FIGURE 2:**
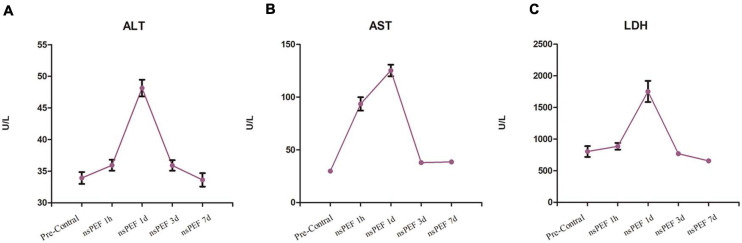
Liver function. The tested liver function results included alanine aminotransferase (ALT; **A**), aspartate aminotransferase (AST; **B**) and lactate dehydrogenase (LDH; **C**). The blood samples for each pig were collected at 1 h pre-treatment and at 1 h, 1 day, 3 days, and 7 days post-treatment.

Biochemical indicators of liver function (ALT, AST, and LDH) reached a peak within 1 day after nsPEF treatment and returned to normal level 7 days after the treatment (Figure 2).

### Metabolomics

A total of 24,037 substance peaks from animal serum, including 8,428 metabolites, were obtained from LC-MS (Supplementary Figure 2). The PCA and OPLS-DA score plots constructed indicated a clear separation between the control vs. 1-h, 1-day, 3-day, and 7-day groups with the resulting peaks (Supplementary Figure 3).

#### Differential Metabolites

**FIGURE 3:**
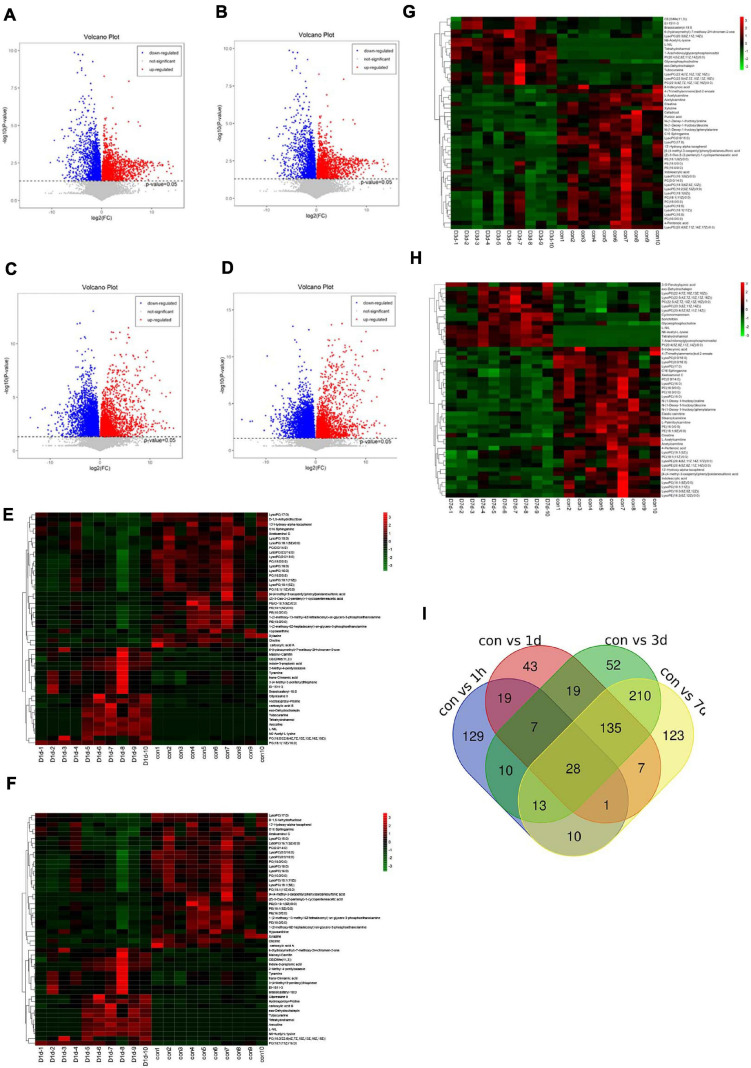
Differential metabolites in serum. Volcano plots show the average normalised quantities of the differentially expressed metabolites in the control and the post-operation groups (**A**: con vs. 1 h, **B**: con vs. 1 day, **C**: con vs. 3 days, **D**: con vs. 7 days). Heat maps show the top 50 differential metabolites in the control groups compared with the post-operation groups (**E**: con vs. 1 h, **F**: con vs. 1 day, **G**: con vs. 3 days, **H**: con vs. 7 days). Carboxylic acid **A**: 3,4,5-trihydroxy-6-[4- (4-methyl-3-oxopentyl)phenoxy]oxane-2-carboxylic acid. Carboxylic acid **B**: 6-{4-[3-(3,7-dimethylocta-2,6-dien-1-yl)-5,7-dihydroxy-6-(4-hydroxy-3-methylbut-2-en-1-yl)-4-oxo-3,4-dihydro-2H-1-benzopyran-2-yl]-3-hydroxyphenoxy}-3,4,5-trihydroxyoxane-2-carboxylic acid. **(I)** A Venn diagram was applied to illustrate that differential metabolites were identified by four cross-comparisons of different postoperative groups with the control group.

We selected serum metabolites with significant differential (*p-*value < 0.05) in the control group relative to the D1h, D1d, D3d, and D7d groups by the VIP values (VIP) > 1.0 of a two-component OPLS-DA model. Figures 3A–D presents volcano plots showing the average normalised quantities of the differentially expressed metabolites in the control groups compared with the post-operative groups. By four cross-comparisons of different post-operative groups with the control group, 259, 217, 474, and 527 differential metabolites were identified (Figure 3I). Figures 3E–H includes heat maps showing the top 50 differential metabolites in the control and the post-operation groups. Subsequently, we selected the top 10 metabolites (Table 1) with the most obvious difference between each group and took the intersection. The disorder of lysophosphatidylcholine (Lyso-PC) and phosphatidylcholine (PC) metabolism were the most obvious among all the paired comparisons, including LysoPC(16:0), LysoPC(0:0/16:0), LysoPC(18:0), LysoPC[18:1(9Z)], LysoPC(16:1(9Z)/0:0), LysoPC(0:0/18:0), LysoPC[18:1(11Z)], PC(18:0/0:0), and PC(16:0/0:0).

**TABLE 1:** Statistical analysis of diagnostic biomarkers: discovery phase.

Metabolites	Retention time (min)	Ratio	VIP value	Fold change	*p*-Value^**†**^	Adjusted *p*-value^‡^
**D1h vs. con**						
PC[16:0/22:6(4Z,7Z,10Z,13Z,16Z,19Z)]	12.245	806.567	66.469	4.634	0.024	0.315
CE[DiMe(11,3)]	3.852	708.632	21.565	1.512	0.016	0.261
*trans*-Cinnamic acid	1.914	166.086	18.205	1.297	0.048	0.422
PC(18:1(11Z)/16:0)	17.436	804.576	13.393	4.556	0.000	0.005
Tyramine	1.929	120.081	9.592	1.303	0.047	0.420
Acetylcarnitine	1.087	204.123	9.534	0.527	0.027	0.336
Sonchifolin	2.352	413.137	9.254	12.548	0.000	0.034
[4-(4-Methyl-3-oxopentyl)phenyl]oxidanesulfonic acid	6.080	271.065	8.333	0.406	0.028	0.343
Pelargonidin 3-sophoroside	8.409	616.176	7.825	3.634	0.009	0.205
Cyclonormammein	3.215	413.137	7.619	7.403	0.001	0.059
**D1d vs. con**						
LysoPC(16:0)	10.063	496.339	52.413	0.583	0.000	0.022
PC(18:0/0:0)	11.090	524.371	38.438	0.626	0.001	0.039
PC[16:0/22:6(4Z,7Z,10Z,13Z,16Z,19Z)]	12.245	806.567	33.150	3.945	0.036	0.252
CE[DiMe(11,3)]	3.852	708.632	24.484	1.791	0.027	0.215
PC(16:0/0:0)	9.859	496.340	19.663	0.510	0.001	0.035
LysoPC(0:0/16:0)	10.073	540.331	19.580	0.693	0.002	0.061
*trans*-Cinnamic acid	1.914	166.086	16.450	1.431	0.025	0.206
LysoPC(18:0)	10.879	524.371	16.247	0.551	0.001	0.041
LysoPC(0:0/18:0)	11.081	568.362	15.621	0.672	0.002	0.059
LysoPC[18:1(9Z)]	10.355	566.347	14.106	0.681	0.010	0.128
**D3d vs. con**						
LysoPC(16:0)	10.063	496.339	51.326	0.626	0.000	0.005
PC(18:0/0:0)	11.090	524.371	27.999	0.773	0.020	0.097
PC(16:0/0:0)	9.859	496.340	19.873	0.527	0.001	0.010
LysoPC(0:0/16:0)	10.073	540.331	16.646	0.766	0.001	0.016
L-Acetylcarnitine	0.748	204.123	16.167	0.148	0.000	0.003
C16 Sphinganine	7.698	274.274	15.082	0.711	0.000	0.006
LysoPC[22:5(4Z,7Z,10Z,13Z,16Z)]	9.968	570.355	15.070	2.877	0.002	0.024
*N*-(1-Deoxy-1-fructosyl)leucine	1.282	294.154	14.732	0.315	0.001	0.011
**D7d vs. con**						
LysoPC(16:0)	10.063	496.339	57.390	0.610	0.000	0.001
PC(18:0/0:0)	11.090	524.371	36.587	0.735	0.001	0.009
PC(16:0/0:0)	9.859	496.340	22.133	0.496	0.000	0.003
LysoPC(0:0/16:0)	10.073	540.331	18.616	0.754	0.000	0.005
L-Acetylcarnitine	0.748	204.123	17.650	0.076	0.000	0.001
C16 Sphinganine	7.698	274.274	16.623	0.697	0.000	0.002
LysoPC(18:0)	10.879	524.371	16.452	0.613	0.000	0.008
*N*-(1-Deoxy-1-fructosyl)leucine	1.282	294.154	14.797	0.314	0.000	0.007
LysoPC[22:5(4Z,7Z,10Z,13Z,16Z)]	9.968	570.355	14.777	2.518	0.000	0.003
LysoPC[18:1(9Z)]	10.355	566.347	14.131	0.755	0.009	0.057

#### Disturbed Metabolic Pathways

**FIGURE 4:**
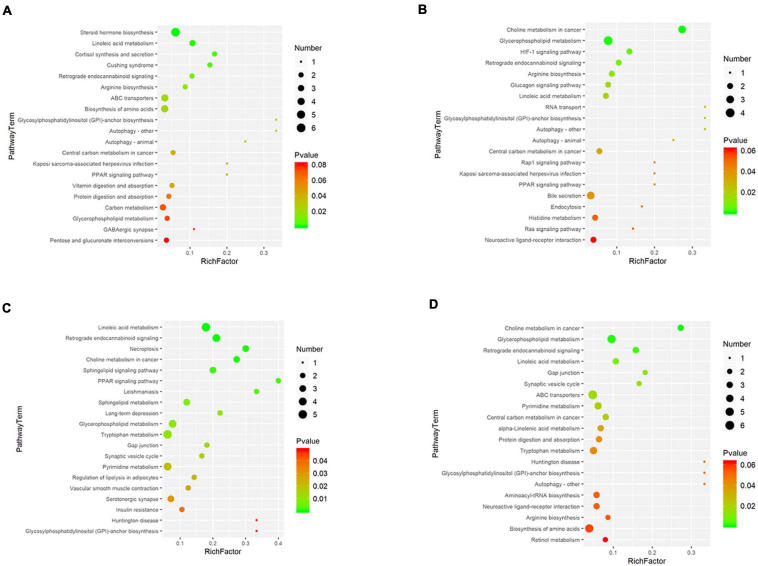
Disturbed metabolic pathways. Bubble charts show the top 20 metabolic pathways that the differentially expressed metabolites enriched in between the pretreatment and the postoperation groups (**A:** con vs. 1 h, **B:** con vs. 1 day, **C:** con vs. 3 days, **D:** con vs. 7 days).

The metabolic pathway enrichment results were obtained based on the Genes and Genomes (KEGG) Pathway Database. The results indicated the different metabolites enriched in metabolic pathways, as follows: glycerophospholipid metabolism, linoleic acid metabolism, glycosylphosphatidylinositol (GPI)–anchor biosynthesis, retrograde endocannabinoid signalling, etc. (Figure 4). Among these pathways, glycerophospholipid metabolism identified the pathway that contributes the most to the metabolic differences.

### Analysis of Gut Microbiota Diversity

**FIGURE 5:**
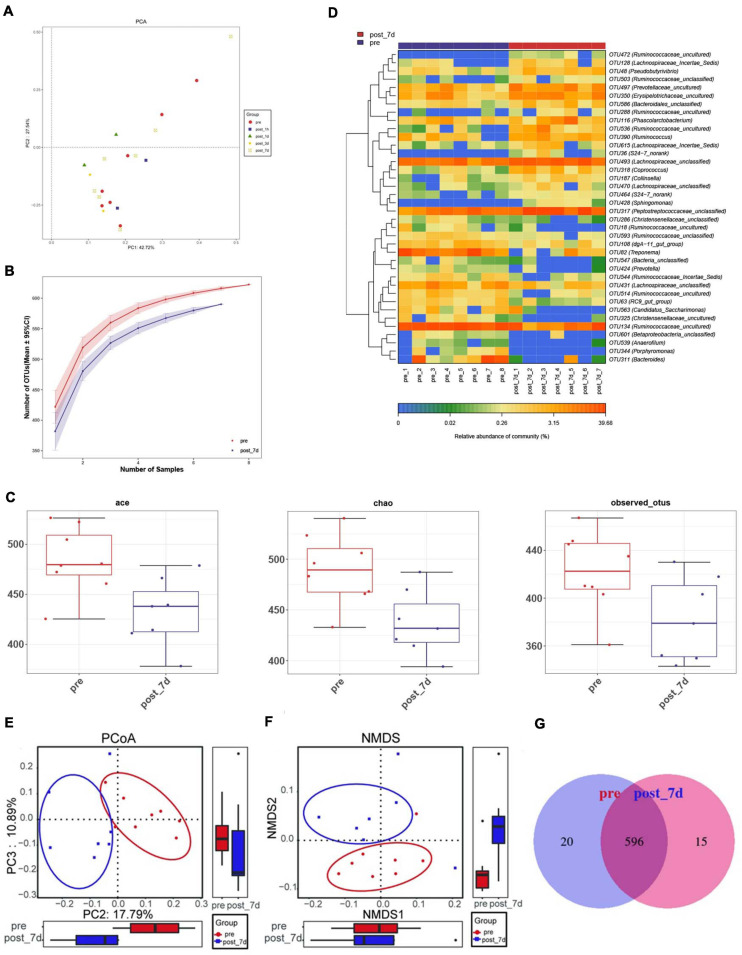
Dynamic changes of faecal microbiota before and after nsPEF treatment. **(A)** Principal coordinate analysis (PCA) of faecal microbiota between the two groups were conducted. **(B)** The rarefaction curves for number of reads sampled between the two groups. **(C)** Comparisons of microbial diversity and richness estimated by Incidence-Based Coverage Estimators (ICE) Index, Chao Index and Observed Species (Obs) Index between the two groups. **(D)** The heat map shows the mean abundances of the prominent OTUs assigned to genus level between before and after nsPEF treatment. **(E)** OPLS-DA score plots show the considerable separation between before and after nsPEF treatment. **(F)** Non-metric multidimensional scaling (NMDS) plot showing alteration in the composition of gut bacterial communities before and after nsPEF treatment. **(G)** A Venn diagram displaying the overlaps between groups showed that 596 of the total richness of 631 OTUs shared between before and after nsPEF treatment.

**FIGURE 6:**
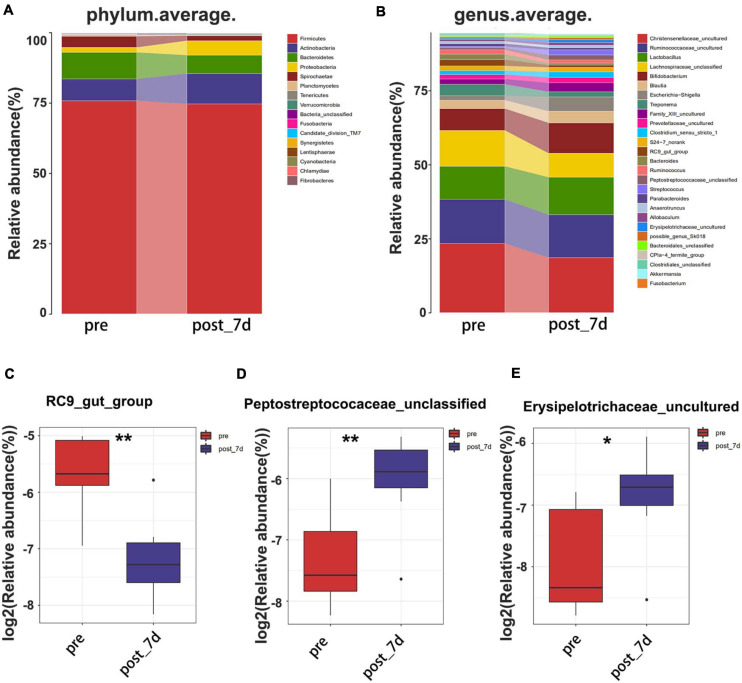
Composition of faecal microbiota assigned to phylum level **(A)** and genus level **(B)** between the two groups. The decreased microbial community **(C)** at the genus level in the post_7d group with the pregroup. The increased microbial community **(D,E)** at the genus level in the post_7d group with the pre-group. The post_7d group, the 7-day group; the pre-group, and the control group.

In order to identify potential factors involved in the alteration of serum metabolites, 16S rRNA gene sequencing was performed to determine the bacterial composition of faecal samples in pigs. The results show the overall structural changes in the intestinal microbial community before and after nsPEF treatment (Figure 5A). The abundance of rare species in intestinal flora decreased significantly after nanosecond ablation (Figures 5B,C). Based on a 97% sequence similarity, an average of 636 OTUs per sample was detected. A total of 38 OTUs were identified as significantly different OTUs before and after nsPEF treatment. Eighteen of these OTUs were decreased, and another 20 OTUs were increased after the 7-day group (the post_7d group) relative to the control group (the pre-group) (Figure 5D). Gut microbiota showed unique characteristics after nsPEF ablation (Figures 5E,F). The Venn diagram intuitively shows the similarity and overlap of OTU number composition in faeces between the 7-day group and the control group (Figure 5G). At the phylum, compared with the pre-group, there were two bacterium with differences in the post_7d group (Figure 6A): Bacteroidetes (*p* < 0.01) downregulated and Fibrobacteres (*p* < 0.05) downregulated. At the genus, compared with the pre-group, there were three bacteria with differences in the post_7d group (Figure 6B): RC9_gut_group (*p* < 0.01) downregulated, Peptostreptococcaceae (*p* < 0.01) upregulated, and Erysipelotrichaceae (*p* < 0.05) upregulated (Figures 6C–E).

### Correlation Analysis Between Serum Metabolism and Gut Microbiota

**FIGURE 7:**
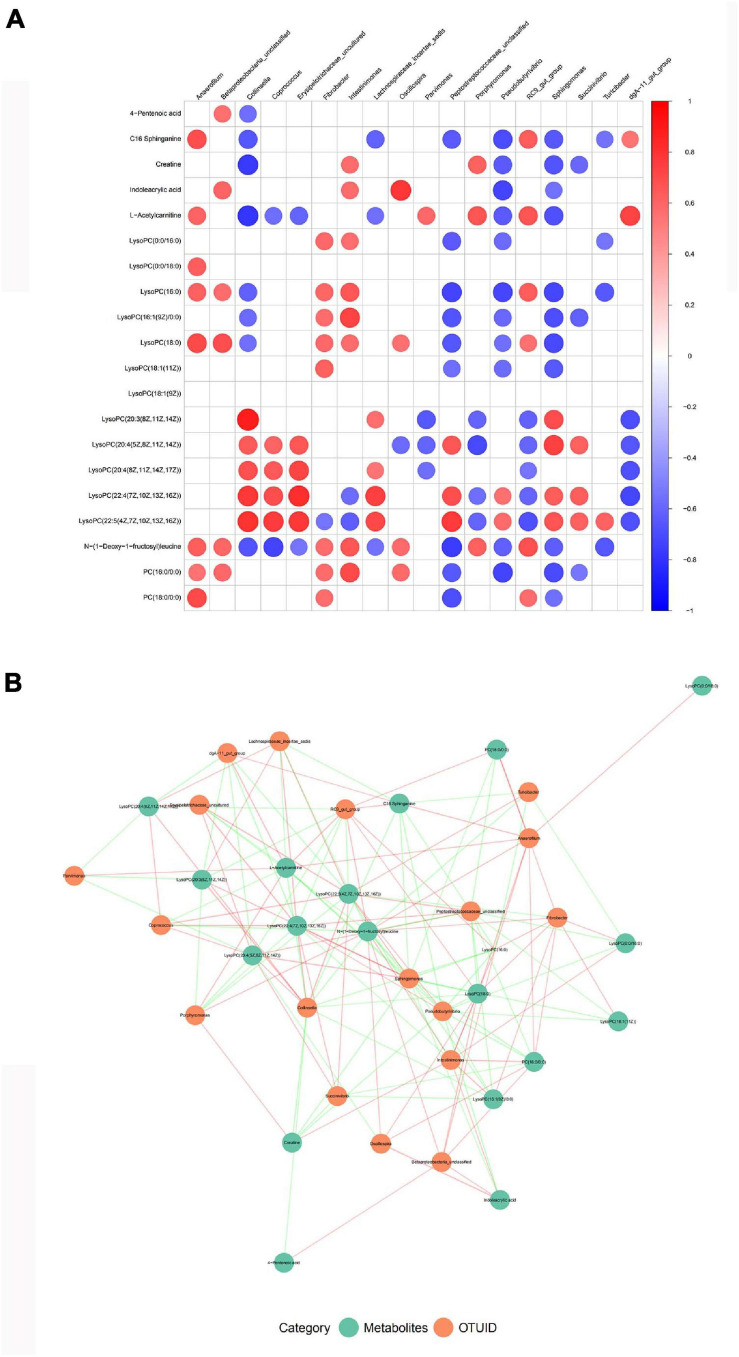
Correlation analysis between gut microbiota and serum metabolism. **(A)** A correlation matrix was drawn for the TOP20 results of correlation analysis of different species and different metabolites. **(B)** Association network diagram of TOP20 differential species and differential metabolites.

Finally, in combination with Spearman’s rank correlation analysis, the relative abundance of gut microbiota was correlated with the relative strength of serum metabolites, so as to find the covariation characteristics of intestinal bacterial structure and overall metabolism of the host. There are important intestinal bacterial members closely related to the changes of serum metabolite pre- and post-treatment (Figure 7A). Some of these bacteria were associated with a variety of changes in blood metabolic abundance, suggesting that these bacteria may affect multiple metabolic pathways during nsPEF treatment (Figure 7B).

## Discussion

Our study describes the change of the serum metabolites and the overall structure of the intestinal microflora before and after the nsPEF treatment, identifying metabolic changes and key flora associated with liver injury after nsPEF ablation. Furthermore, a preliminary interpretation of the potential significance and association of these changes provides the underlying data for the safety and feasibility of nsPEF in liver applications.

It is well known that the liver is an important site for lipid metabolism, including the metabolism of triacylglycerol (TAG) and other lipids, such as phospholipids, cholesterol, and free fatty acids. Lyso-PC is a product of glycerolipid metabolism. Lyso-PC with high abundance has important physiological function and activity in organism. Several studies have shown reduced total LysoPC and multiple LysoPC molecules in the blood of obese and T2DM patients (Barber et al., 2012). PC is one of the major phospholipid components of all plasma lipoprotein classes. Targeted or untargeted metabolomics and lipid omics studies have shown that LysoPC (18:0), LysoPC(18:2), LysoPC(20:4), LysoPC (O-22:1), etc., can distinguish healthy individuals from patients with T2DM, thereby achieving their early risk prediction (Zhu et al., 2011; Mamtani et al., 2016; Tonks et al., 2016). PC is the only phospholipid known to be required for the assembly and secretion of lipoprotein. Impaired hepatic PC biosynthesis can significantly reduce the levels of circulating high-density lipoproteins (HDLs) and very low-density lipoproteins (VLDLs). Existing research shows that the metabolic abnormalities of lipid content can cause many human diseases, including metabolic diseases such as diabetes (Antwi-Baffour et al., 2018), fatty liver (Ipsen et al., 2018), obesity (Klop et al., 2013), cardiovascular disease (Miname and Santos, 2019), atherosclerosis (Zhao et al., 2018) and hypertension (Sander and Giles, 2002), cancer diseases such as endometrial cancer (Shi et al., 2019), leukaemia (Morel et al., 2017), prostate cancer (Chavarro et al., 2013; Xin et al., 2016), and liver cancer (Wang et al., 2016). In our experiments, biochemical indicators of liver have returned to normal 7 days after nsPEF ablation, but the change of the metabolites still exists. This phenomenon indicates that although the clinical indicators return to normal after the nsPEF ablation, other risks may still exist, which plays a certain warning role for the application of this technology. If the technology is applied clinically, more researches are needed to determine whether such metabolic changes occur and whether they affect patients in the long term.

Gut microbiota plays different physiological functions in the metabolism of the body and maintains a dynamic balance with the intestinal environment. Once this balance is broken, it will lead to corresponding diseases (Kuno et al., 2016). There is growing evidence that gut bacteria can influence liver-related diseases, and that microbial dysfunction is associated with the development of a variety of liver diseases, such as non-alcohol fatty liver disease (NAFLD). Compared with healthy people, the overview of bacteria in the gut flora of NAFLD patients shows distinctive features that may predict NAFLD (Wu et al., 2015). A recent study showed that oral sulforaphane alleviated hepatic steatosis in mice with a long-term high-fat diet by suppressing inflammatory signals in NLRP3. This suggests that gut microbiota may be associated with NAFLD progression through innate immune mechanisms (Bach Knudsen, 2015; Yang et al., 2016). Some scholars explored the relationship between liver cancer and gut microbiota through animal models, and found that helicobacter hepatis in gut microbiota can increase the risk of liver cancer by enhancing the virulence of aflatoxin (Raman et al., 2013). In addition, gut microbiota has also been reported correlating with the development of liver diseases such as liver cirrhosis (Bajaj et al., 2015; Chen et al., 2016) and cholestatic liver disease (Iwasawa et al., 2017; Kummen et al., 2017). All the studies above are conducted from the perspective of the influence of bacterial imbalance on the occurrence and development of liver diseases. From the perspective of the effect of liver injury on gut microbiota, we conducted a relatively novel and comprehensive evaluation of the safety and feasibility of this technique.

Although we preliminarily understand the structural changes of intestinal bacterial community during liver regeneration through 16S rRNA sequencing, we are still faced with the following question: What specific functions do these microbial communities have? As we know, gut microbiota is involved in important physiological processes such as food digestion, nutrient metabolic absorption, drug metabolism, energy supply, production of essential vitamins, immune regulation, and maintenance of gastrointestinal homeostasis. The development of gene sequencing technology and bioinformatics has provided more powerful tools for the study of intestinal flora, which can not only quantify and classify bacteria from the level of bacteria genera but also enables us to deeply understand the characteristics of bacteria in metabolism and other aspects through functional gene comparison. Scientists have successively established their own intestinal metagenomes and elaborated the classification and function of intestinal microorganisms (Ren et al., 2018, 2019). This predictive metagenomics approach will provide a new functional approach for the study of thousands of microorganisms that have not yet been able to conduct tissue culture. The results of gene prediction in this study showed that most of the functional genes represented by gut microbiota were related to metabolism (Supplementary Figure 4), which is consistent with the serum metabolic changes we found, but the mechanism remains to be further studied.

## Data Availability Statement

The Metabolic data presented in the study are deposited in the (Molecular Biology Laboratory’s European Bioinformatics Institute) repository, accession number (MTBLS2406). The data for all faecal samples presented in the study are deposited in the (National Center for Biotechnology Information Sequence Read Archive database) repository, accession number (PRJNA730351).

## Ethics Statement

The animal study was reviewed and approved by Ethical Committee of Zhejiang Chinese Medical University Laboratory Animal Research Center (approval number IACUC-20191216- 04).

## Author Contributions

YD and JL contributed in conducting the experiments, analysis and interpretation of data, and drafting of the manuscript. XC improved the design of the study and provided the nsPEF equipment. ZR, LH, and HW collected the samples and analysed the related data. TW, ZH, and DY contributed in conducting the experiments. HX and WZ conceived and supervised the project. All authors contributed to the article and approved the submitted version.

## Conflict of Interest

The authors declare that the research was conducted in the absence of any commercial or financial relationships that could be construed as a potential conflict of interest.
