# Supplementary figures and images for: Multi-Omics Analysis Reveals Disturbance of Nanosecond Pulsed Electric Field in the Serum Metabolic Spectrum and Gut Microbiota
Source: Front Microbiol. 2021 Jul 2;12:649091. doi: 10.3389/fmicb.2021.649091 (PMC8283677; doi:10.3389/fmicb.2021.649091)

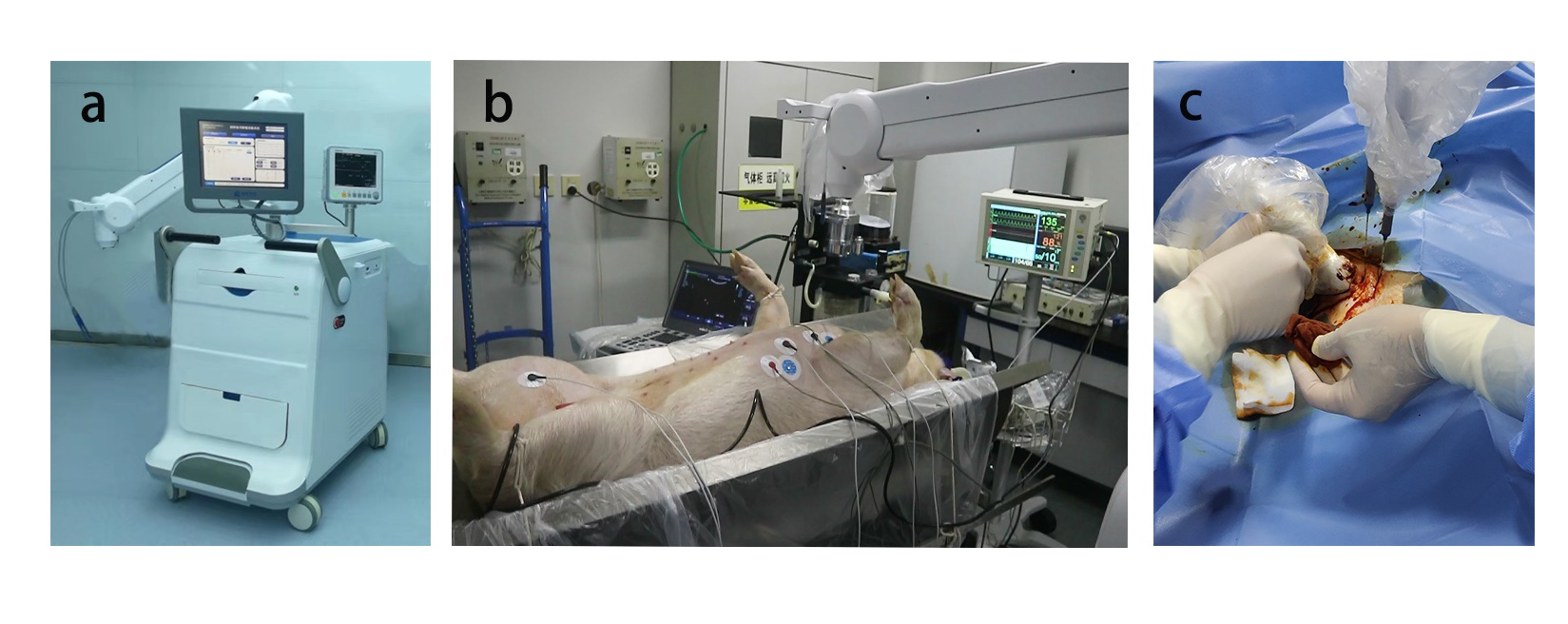

Supplement: Supplementary file 1 [file Image_1.JPEG]

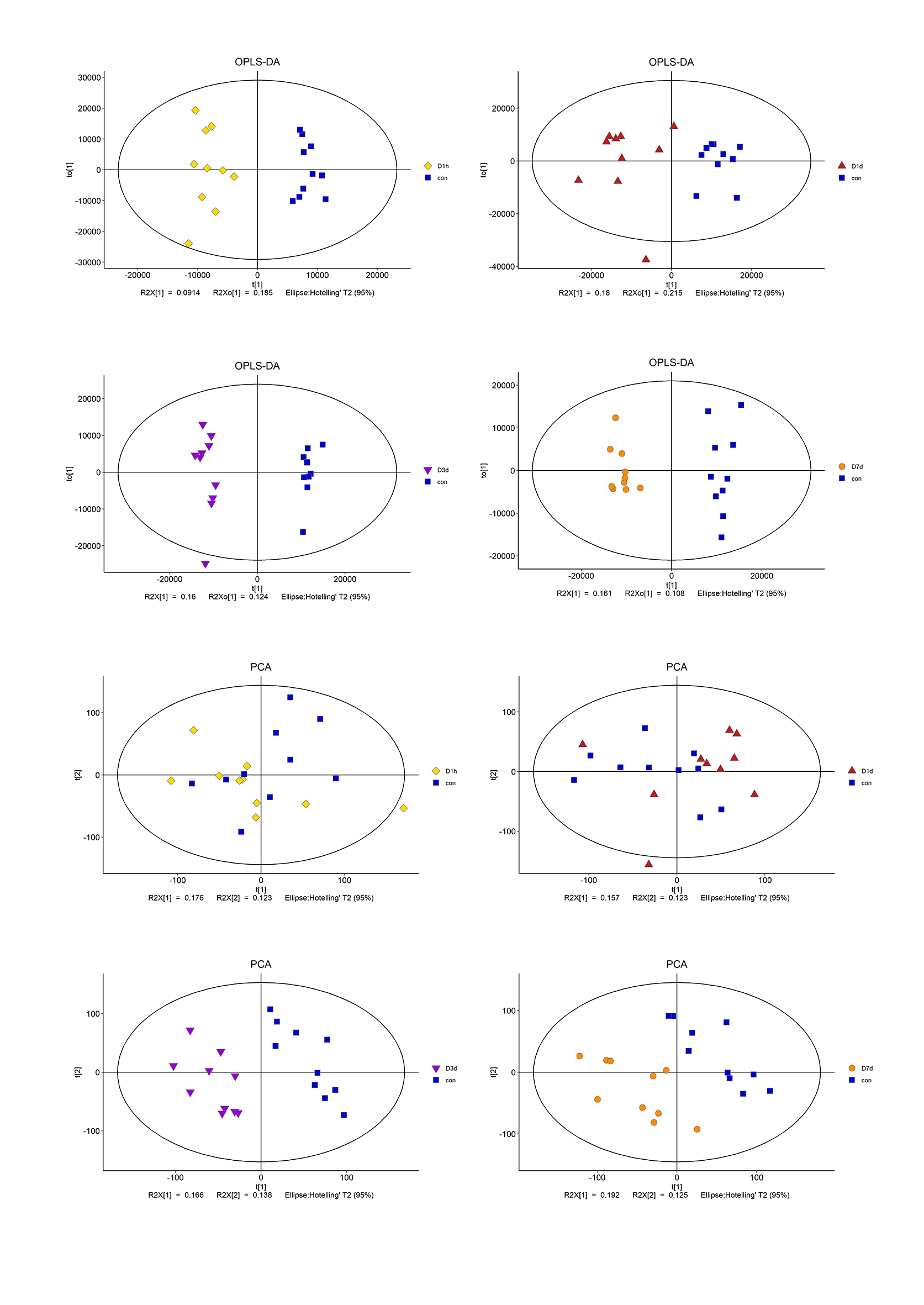

Supplement: Supplementary file 2 [file Image_2.TIF]

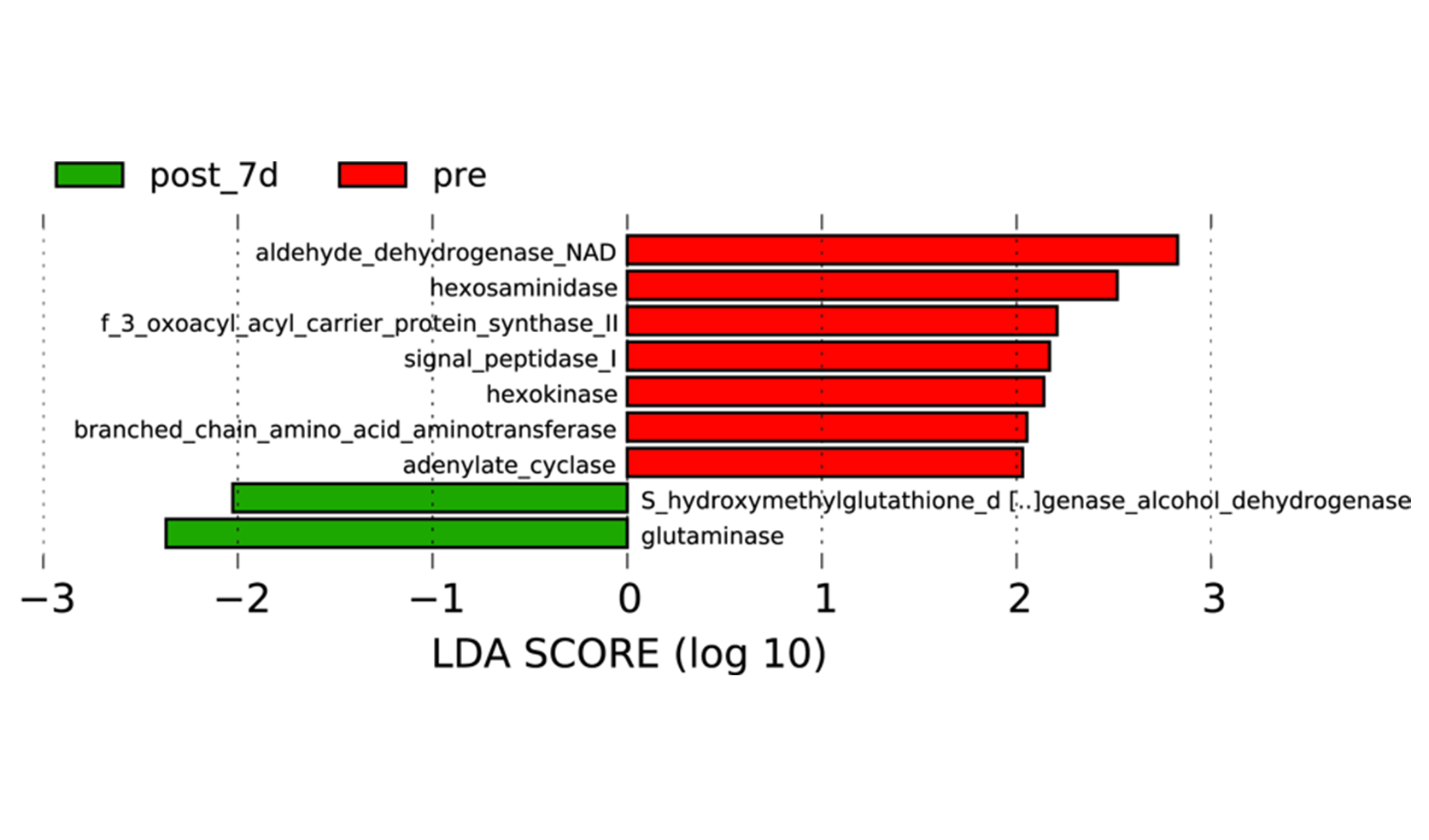

Supplement: Supplementary file 3 [file Image_3.TIF]

Neg:

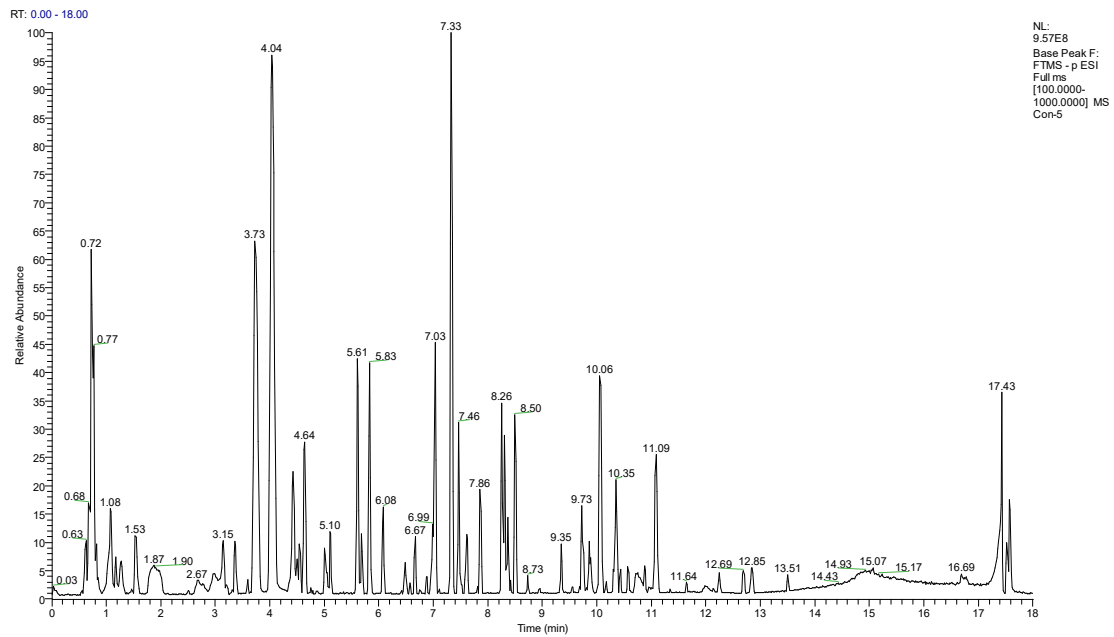

con

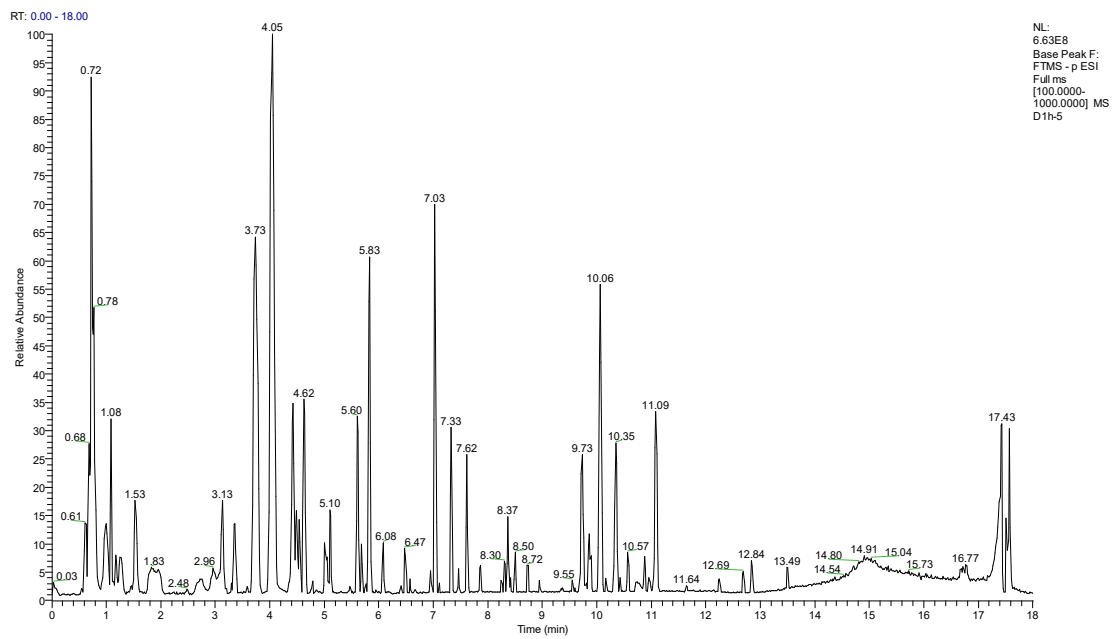

D1h

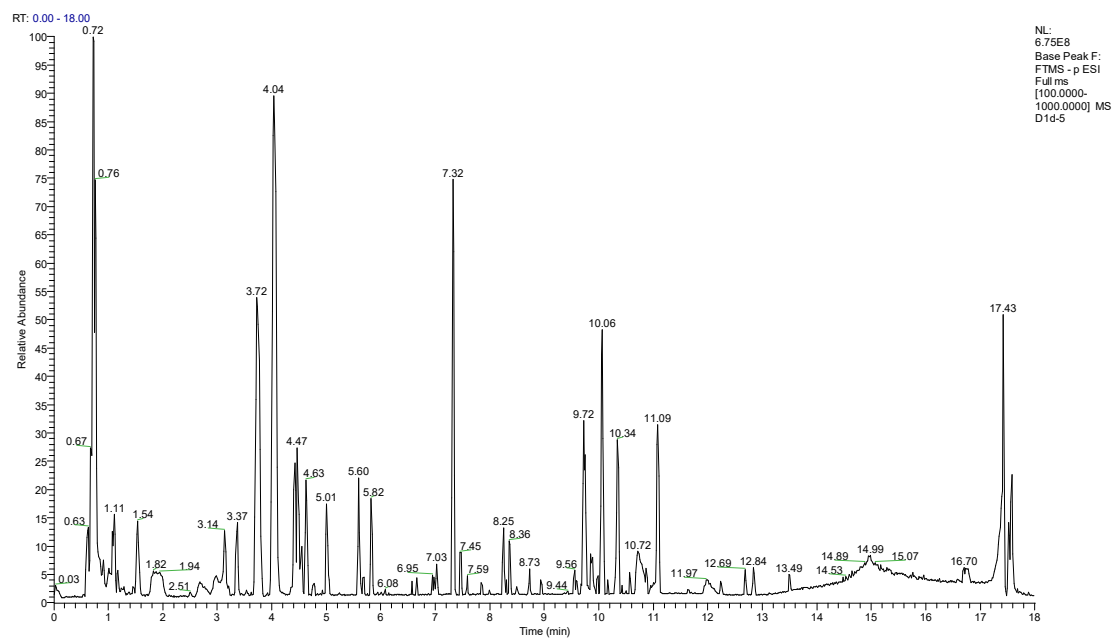

D1d

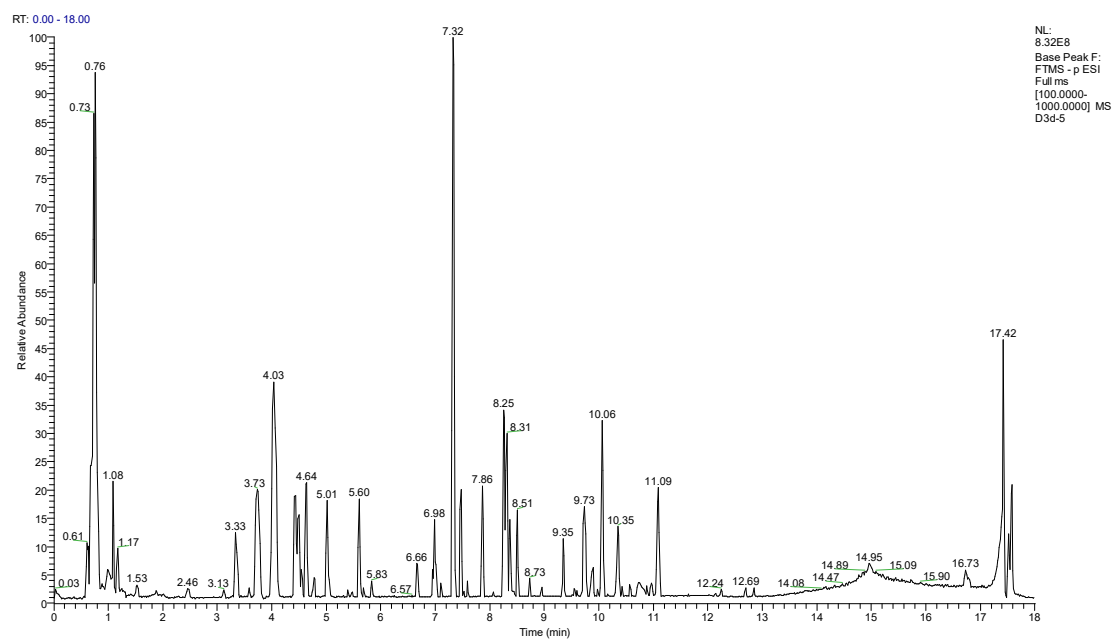

D3d

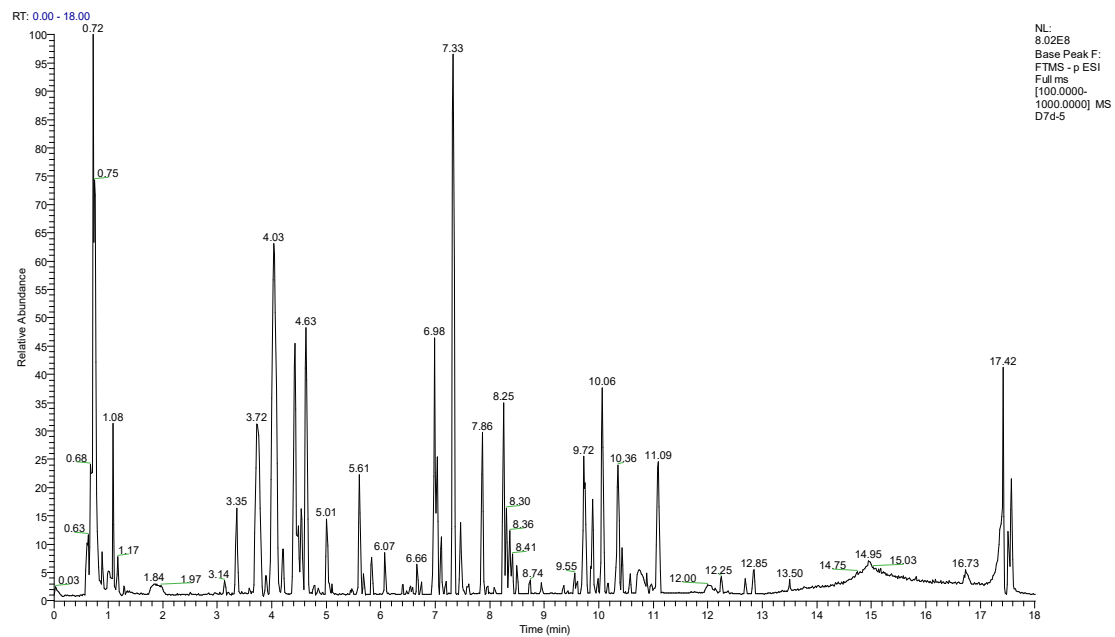

D7d

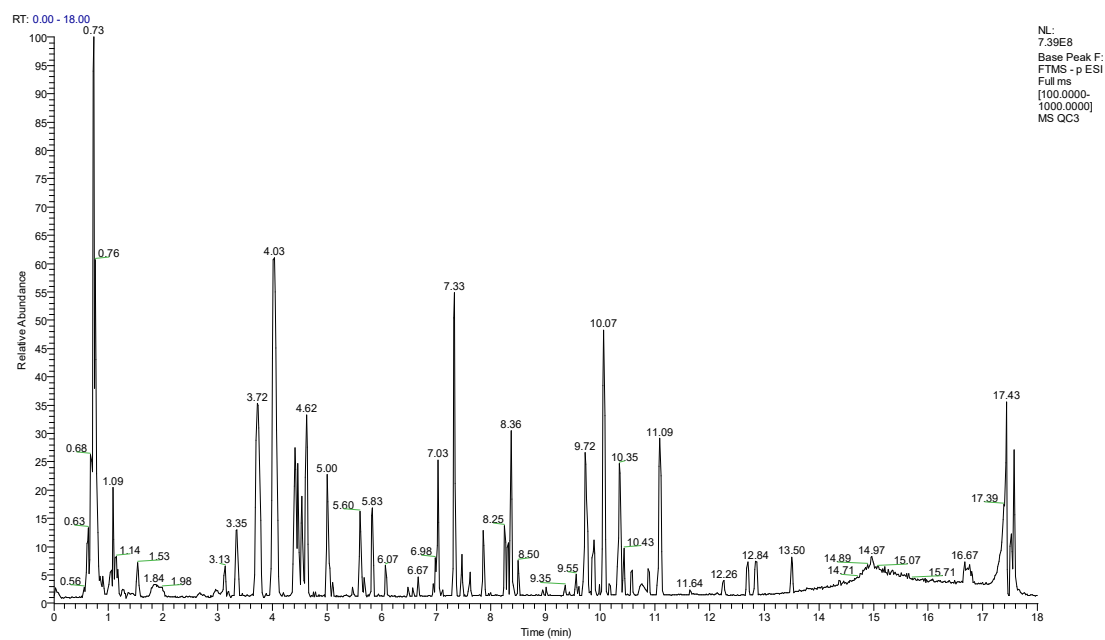

QC

pos:

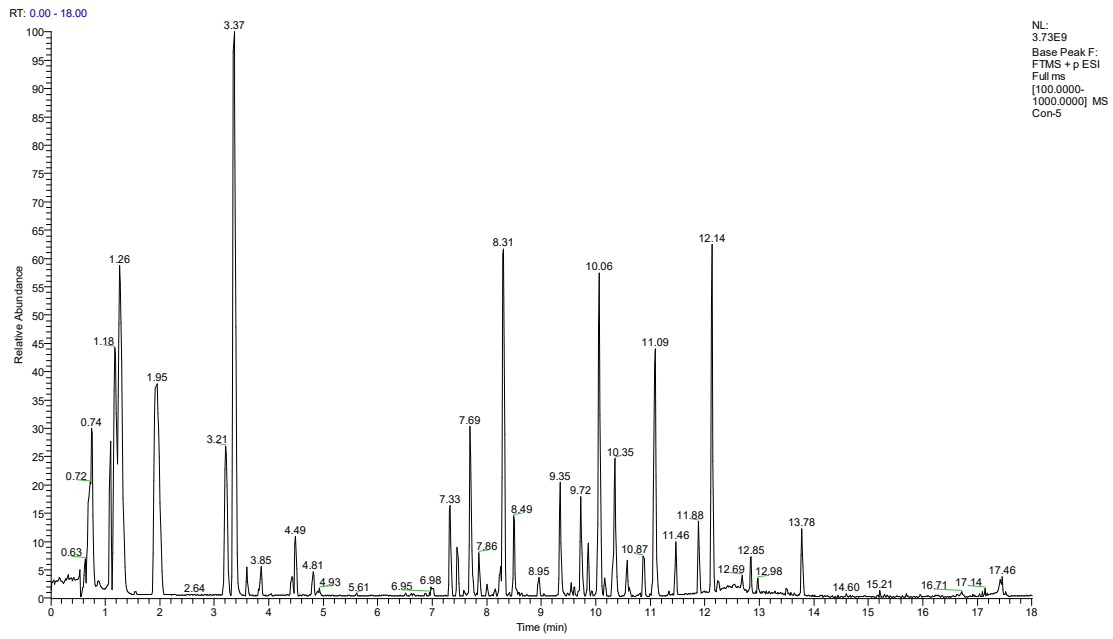

con

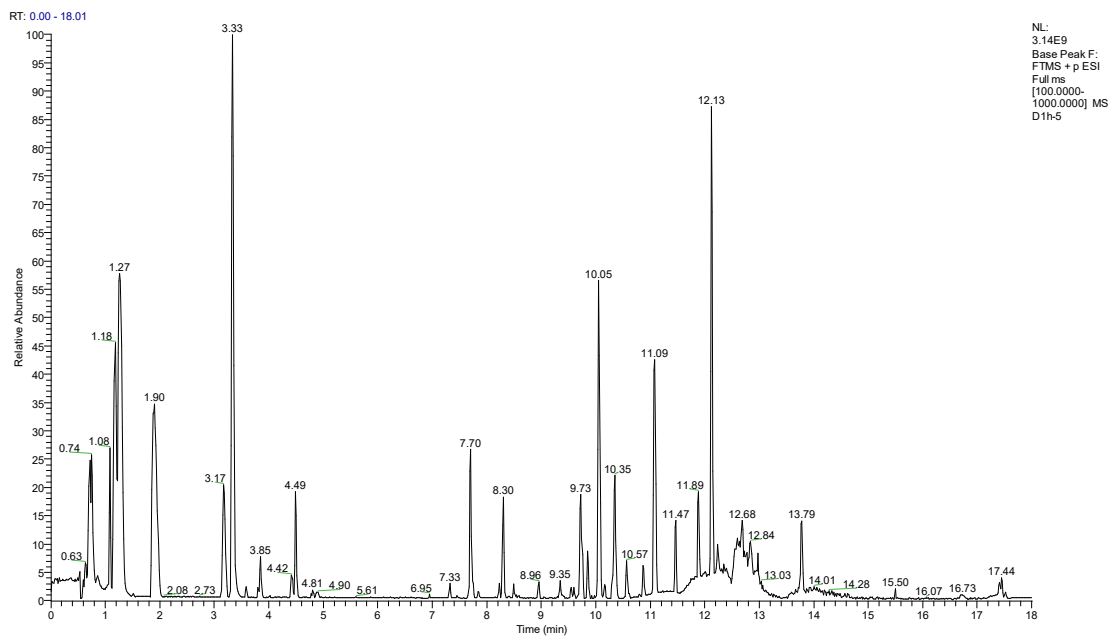

D1h

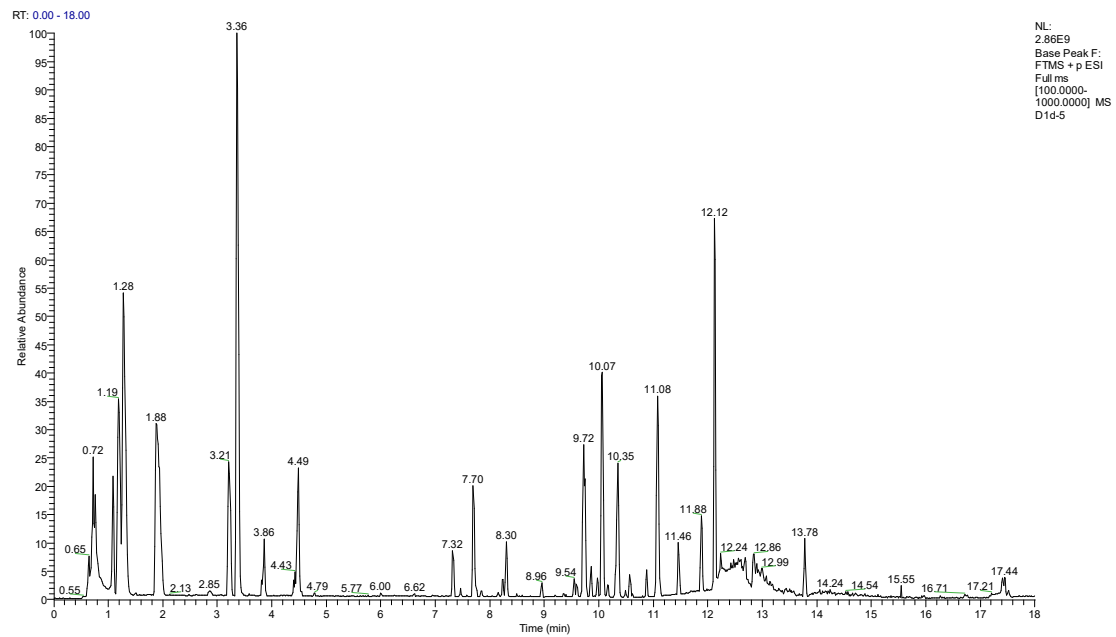

D1d

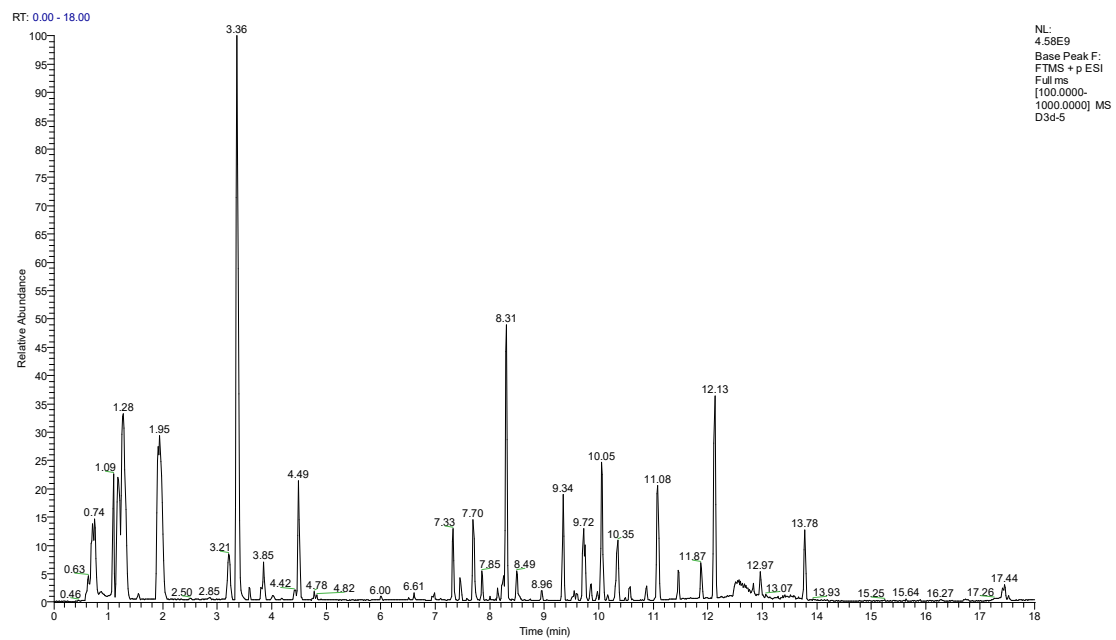

D3d

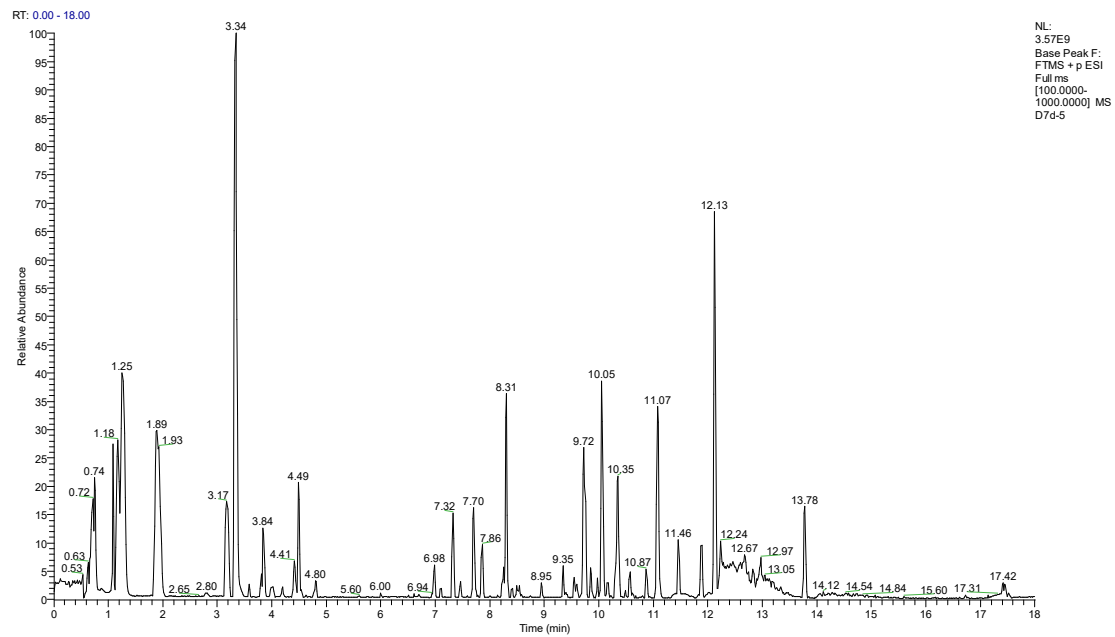

D7d

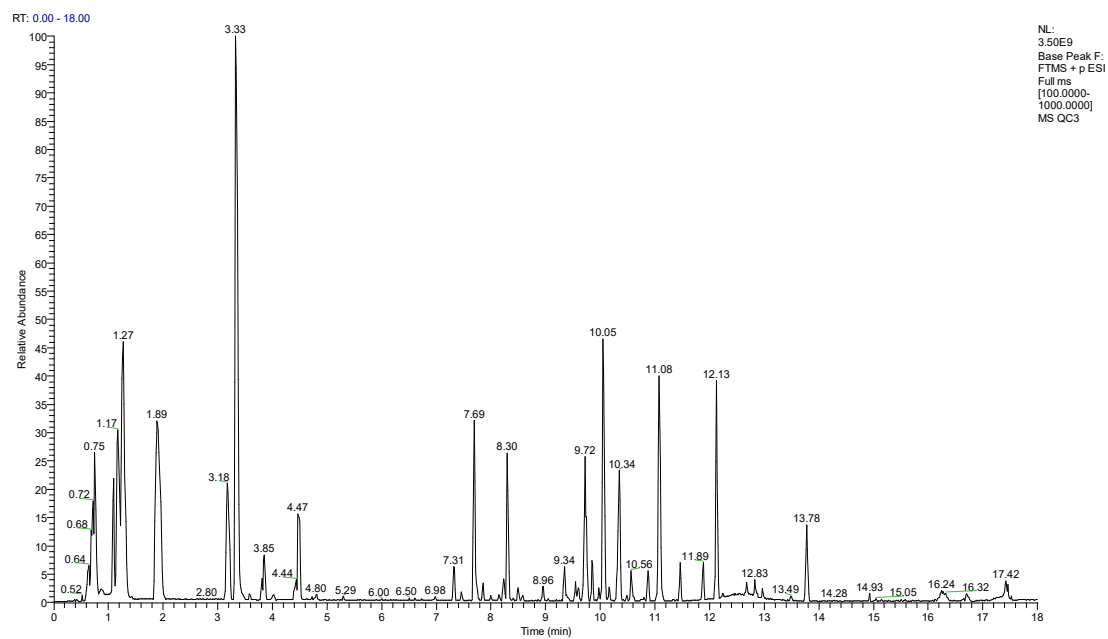

QC

Supplement: Supplementary file 4 [file Data_Sheet_1.PDF]
